# A Comparative Study on Phytochemical Profiles and Biological Activities of *Sclerocarya birrea* (A.Rich.) Hochst Leaf and Bark Extracts

**DOI:** 10.3390/ijms19010186

**Published:** 2018-01-08

**Authors:** Daniela Russo, Rocchina Miglionico, Monica Carmosino, Faustino Bisaccia, Paula B. Andrade, Patrícia Valentão, Luigi Milella, Maria Francesca Armentano

**Affiliations:** 1 University of Basilicata, Department of Science, V.le dell’AteneoLucano, 85100 Potenza, Italy; daniela.russo@unibas.it (D.R.); rocchina.miglionico@virgilio.it (R.M.); monica.carmosino@unibas.it (M.C.); faustino.bisaccia@unibas.it (F.B.); mariafrancesca.armentano@unibas.it (M.F.A.); 2 REQUIMTE/LAQV, Laboratório de Farmacognosia, Departamento de Química, Faculdade de Farmácia, Universidade do Porto, R. Jorge Viterbo Ferreira, nº 228, 4050-313 Porto, Portugal; pandrade@ff.up.pt (P.B.A.); valentao@ff.up.pt (P.V.)

**Keywords:** polyphenols, cytotoxic effect, ROS, apoptosis, mitochondrial membrane potential, HepG2 cell line

## Abstract

*Sclerocarya birrea* (A.Rich.) Hochst (Anacardiaceae) is a savannah tree that has long been used in sub-Saharan Africa as a medicinal remedy for numerous ailments. The purpose of this study was to increase the scientific knowledge about this plant by evaluating the total content of polyphenols, flavonoids, and tannins in the methanol extracts of the leaves and bark (MLE and MBE, respectively), as well as the in vitro antioxidant activity and biological activities of these extracts. Reported results show that MLE is rich in flavonoids (132.7 ± 10.4 mg of quercetin equivalents/g), whereas MBE has the highest content of tannins (949.5 ± 29.7 mg of tannic acid equivalents/g). The antioxidant activity was measured using four different in vitro tests: β-carotene bleaching (BCB), 2,2′-azino-bis(3-ethylbenzothiazoline-6-sulfonic acid) (ABTS), O_2_^−•^, and nitric oxide (NO^•^) assays. In all cases, MBE was the most active compared to MLE and the standards used (Trolox and ascorbic acid). Furthermore, MBE and MLE were tested to evaluate their activity in HepG2 and fibroblast cell lines. A higher cytotoxic activity of MBE was evidenced and confirmed by more pronounced alterations in cell morphology. MBE induced cell death, triggering the intrinsic apoptotic pathway by reactive oxygen species (ROS) generation, which led to a loss of mitochondrial membrane potential with subsequent cytochrome c release from the mitochondria into the cytosol. Moreover, MBE showed lower cytotoxicity in normal human dermal fibroblasts, suggesting its potential as a selective anticancer agent.

## 1. Introduction

Plants are considered to be producers of natural compounds that have been used by humans in medicines to treat diseases since ancient times. Nowadays, a large number of drugs used against various diseases have been isolated or derived from natural sources, often starting from their use in traditional medicine [1].

The biological activities from natural sources are due to the variety of chemicals that are widely distributed in nature [2,3,4,5]. In particular, several studies have attributed important bioactivities to polyphenols, which include anti-inflammatory, analgesic, antimicrobial, and apoptosis-inducing properties, and the possible use of these compounds as anticancer drugs [6,7]. It has been previously demonstrated that polyphenols can act as both antioxidants and pro-oxidants, depending on their concentration and the cellular environment [8].

*Sclerocarya birrea* (A.Rich.) Hochst (Anacardiaceae), commonly known as marula, has long been used in sub-Saharan Africa as a medicinal remedy for numerous ailments: indeed, the stem bark, roots, and leaves of *S. birrea* are used to treat hypertension, diabetes, dysentery, and inflammation [9].

It has been reported that extracts from *S. birrea* roots inhibit the growth of *Candida* spp. and *Cryptococcus neoformans* [10]; the root extract also possesses important in vitro antioxidant activity with particular regard to its free radical scavenging activity, and exhibits cytotoxic effects linked to increasing amounts of reactive oxygen species (ROS) in HepG2 cells [11,12]. Moreover, water and acetone extracts of stem bark show anticancer and proapoptotic activities [13]. Previous studies [14,15,16] investigated the phytochemical profile of different parts of *S. birrea* and it was found that polyphenols, including phenolic acid, flavonoids and their glycosides and procyanidins, were the main compounds. Considering the antioxidant and proapoptotic activity of the methanol extract of *S. birrea* root [12] and in order to clarify the biological activity of different parts of this plant, the aim of this study was to investigate the in vitro antioxidant properties and the cytotoxic activity of leaf and bark methanol extracts in a carcinoma cell line (HepG2). To the best of our knowledge, this is the first study that has evaluated and compared the cytotoxic activities of *S. birrea* bark and leaf extracts on the human hepatocarcinoma cell line (HepG2) vs. fibroblasts.

## 2. Results

### 2.1. Total Polyphenol (TPC), Flavonoid (TFC), and Tannin Content (TTC) Evaluation

Table 1 shows the total polyphenol content (TPC) of the extracts measured using the Folin–Ciocalteu reagent; the values are expressed as milligram (mg) of gallic acid equivalents (GAE) per gram (g) of dried extract. The TPC values of the *S. birrea* leaf extracts ranged from 30.2 ± 1.3 mg GAE/g (*n*-hexane leaf extract, HLE) to 62.6 ± 0.8 mg GAE/g (methanol leaf extract, MLE), whereas the TPC values of the bark extracts ranged from 31.3 ± 0.2 mg GAE/g (chloroform:methanol bark extract, CMBE) to 241.3 ± 8.9 mg GAE/g (methanol bark extract, MBE). The tannin content of the leaf and bark extracts are expressed as milligram (mg) of tannic acid equivalents (TAE) per gram (g) of dried extract and ranged from 74.2 ± 5.5 mg TAE/g (chloroform:methanol leaf extract, CMLE) to 175.8 ± 5.5 mg TAE/g (CLE) in *S. birrea* leaves and from 158.7 ± 1.6 mg TAE/g (CMBE) to 949.5 ± 29.7 mg TAE/g (MBE) in bark extracts. The highest TFC (mg of quercetin equivalents (QE) per g) was observed in MLE (132.7 ± 10.4 mg QE/g of dried extract) and MBE (57.7 ± 3.5 mg QE/g of dried extract), whereas the lowest values were observed in *n*-hexane extracts (8.3 ± 1.2 mg TAE/g in HLE and 4.7 ± 0.5 mg TAE/g in *n*-hexane bark extract (HBE)) (Table 1).

### 2.2. Antioxidant Activity

**Figure 1:**
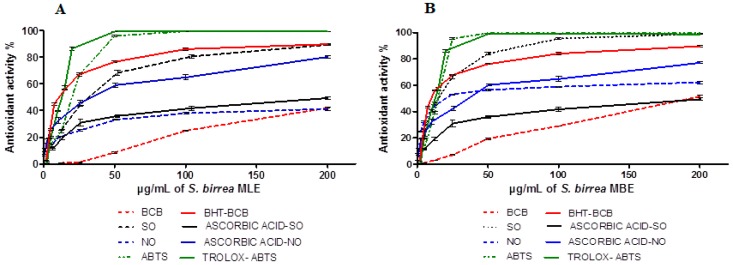
Antioxidant activity in vitro. Dose-dependent antioxidant activity of (**A**) *S. birrea* methanol bark extract (MBE) and (**B**) leaf extract (MLE) compared with the reference standards. Antioxidant activity was determined by four assays: 2,2′-azino-bis(3-ethylbenzothiazoline-6-sulfonic acid) (ABTS), β-carotene bleaching (BCB), superoxide anion (SO), and nitric oxide (NO).

All the extracts were tested to evaluate their antioxidant activity and preliminary assays indicated that the methanol extracts were more active than the others (Material not intended for publication: [17]). Antioxidant activity was measured by four spectrophotometric tests. Inhibition of lipid peroxidation was carried out by the β-carotene bleaching (BCB) assay and butylated hydroxytoluene (BHT) was used as the standard (IC_50_ = 11.3 ± 0.2 µg/mL). The inhibition was dose dependent (Figure 1A,B) and methanol extracts showed a moderate inhibition with an IC_50_ value of 197.1 ± 14.2 µg/mL for MBE, whereas MLE was not able to overcome the IC_50_ even at the highest tested concentration. In vitro assays can be used to monitor the ability of plant extracts to quench radicals and in this study radical scavenging activity was monitored against synthetic (2,2′-azino-bis(3-ethylbenzothiazoline-6-sulfonic acid) (ABTS) radical) and physiological radicals (nitric oxide radical and superoxide anion). Methanol extracts showed a high dose-dependent ABTS scavenging activity (Figure 1A,B). At the highest concentration (200 µg/mL), both methanol extracts showed about 100% scavenging activity; thus, the IC_50_ value is needed to better understand the slight differences between the investigated vegetal parts. The IC_50_ calculated for the leaves and bark were 18.7 ± 1.5 and 15.0 ± 0.9 µg/mL, respectively. Trolox was used as the standard and its IC_50_ was 12.8 ± 0.9 µg/mL, evidencing the strong antioxidant potential of the extracts.

Both vegetal parts showed antiradical activity against the investigated physiological radicals in a dose-dependent manner (Figure 1A,B), but MBE was more able to quench both radicals than MLE. In particular, MBE showed IC_50_ values of 19.9 ± 2.9 and 15.6 ± 1.0 µg/mL for nitric oxide and superoxide anion, respectively. MLE showed an IC_50_ value of 31.1 ± 3.3 µg/mL for superoxide anion, but a low inhibition of nitric oxide reaching 39.9 ± 0.5% inhibition at 200 µg/mL. In both assays, ascorbic acid was used as the standard showing IC_50_ values of 35.6 ± 2.4 and 199.3 ± 20.1 µg/mL for nitric oxide and superoxide anion, respectively.

### 2.3. Cytotoxic Effect of Bark and Leaf Methanol Extracts

Using the Calcein AM viability assay, the cytotoxic effects of MBE and MLE from *S. birrea* were tested on HepG2 and normal human dermal fibroblast cells treated with different concentrations of both extracts for 24 h.

**Figure 2:**
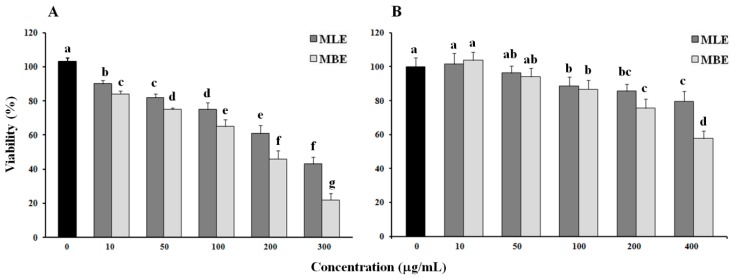
Cell viability assay. Cytotoxic effects of the methanol extract from leaves (MLE) and methanol extract from bark (MBE) of *S. birrea* on HepG2 cells (**A**) and human dermal fibroblasts (**B**). Cells were exposed to the indicated concentrations of MLE and MBE for 24 h; cell viability was then evaluated with the Calcein AM assay as described in the Materials and Methods section. Values are the mean ± SD of three replicates from three independent experiments. Significant differences (*p* < 0.05) are highlighted with different letters.

As indicated in Figure 2A, MBE exhibited a dose-dependent cytotoxic activity on the human cancer cell line, which was higher than MLE (IC_50_ = 180 and 270 µg/mL, respectively), although its activity was lower than that of the methanol extract from roots (MRE) that was previously demonstrated [12]. 

Both extracts exhibited lower toxicity towards normal cells (IC_50_ > 400 µg/mL), suggesting their use as a potential anticancer agent (Figure 2B).

### 2.4. Cell Morphology Analysis

**Figure 3:**
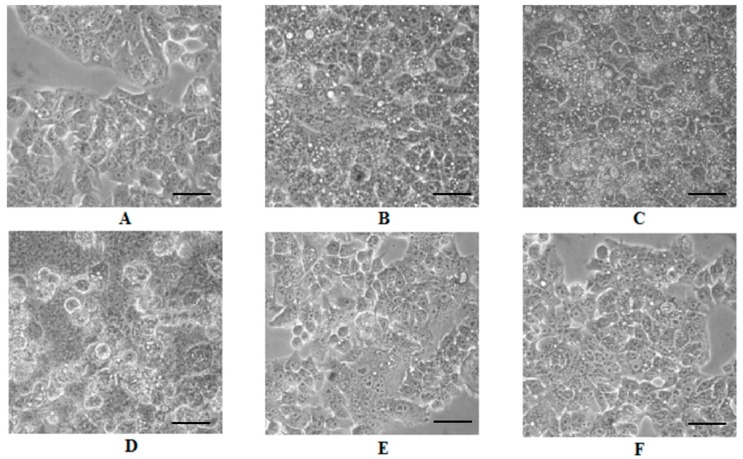

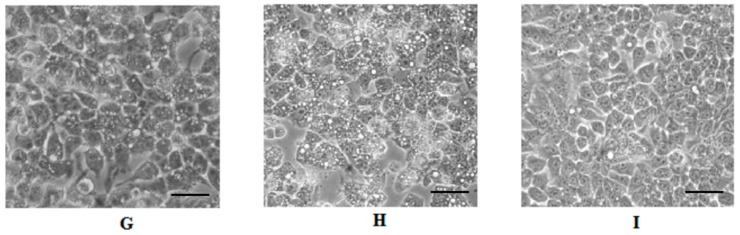
Morphological cell analysis. Inverted phase contrast images showing changes in HepG2 cells induced by leaf (MLE) and bark (MBE) methanol extracts of *S. birrea*. (**A**) 10 µg/mL MBE; (**B**) 50 µg/mL MBE; (**C**) 100 µg/mL MBE; (**D**) 200 µg/mL MBE; (**E**) 10 µg/mL MLE; (**F**) 50 µg/mL MLE; (**G**) 100 µg/mL MLE; (**H**) 200 µg/mL MLE; (**I**) control cells. Scale bars: 100 µm.

Morphological alterations of HepG2 cells treated with both extracts were observed under a phase contrast microscope. MBE-treated cells showed dose-dependent morphological alterations: many cytoplasmic vacuoles were observed, which progressively increased in number and size in proportion to the MBE concentration. Moreover, at 200 µg/mL, the majority of cells lost their typical morphology and appeared round and translucent (Figure 3D). In the case of MLE, the effects of the treatment were much less severe: a strong vacuolization with few dead cells only at 200 µg/mL (Figure 3H) when compared with untreated HepG2 cells (Figure 3I).

### 2.5. Evaluation of Apoptosis in HepG2 Cells Treated with MLE and MBE

**Figure 4:**
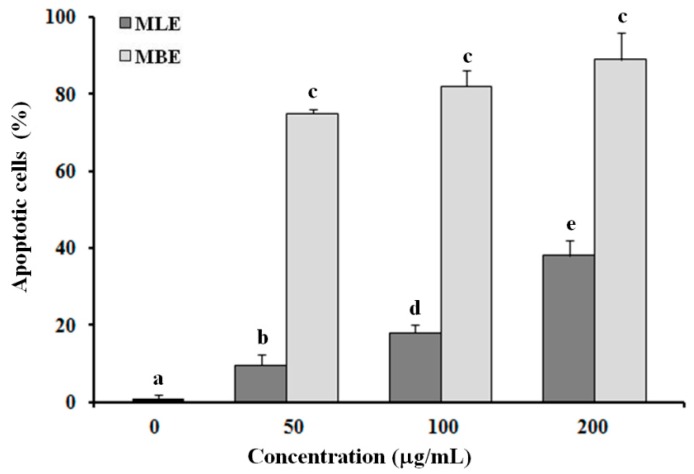
Quantitative evaluation of apoptosis by a flow cytometry assay. HepG2 cells were incubated for 24 h with 50, 100, and 200 µg/mL of MLE or MBE and apoptosis was measured using flow cytometry after Annexin V/7-AAD double staining. Values are the mean ± SD of three replicates from three independent experiments. Significant differences (*p* < 0.05) are highlighted with different letters.

The apoptotic effects of MLE and MBE were evaluated in HepG2 cells exposed to different concentrations (50, 100 and 200 µg/mL) of each extract for 24 h; cells were analyzed by flow cytometry after staining with Annexin V/7-AAD (Figure 4). Untreated cells showed a low percentage of dead cells (about 1%), while the number of Annexin V/7-AAD-positive cells were prominent in MBE treated cells, indicating the involvement of an apoptotic process in cell death. The extract induced apoptosis in a dose-dependent manner (the percentage of apoptotic cells: 75.2 ± 1.4%, 82.0 ± 5.3%, and 89.0 ± 7.2% at 50, 100, and 200 µg/mL, respectively), showing a trend similar to that of MRE [12]. In contrast, MLE showed lower apoptotic activity than that of MBE and MRE: after treatment with leaf extracts, the proapoptotic rate increased to 9.5 ± 3.1%, 18.2 ± 2.3%, and 38.4 ± 4.2% in a dose-dependent manner.

### 2.6. Effects of MLE and MBE on Both ROS Production and Mitochondrial Membrane Potential (ΔΨ_m_)

To investigate the role of ROS in extract-mediated apoptosis, we determined ROS production in HepG2 cells after 3 h of treatment with MLE or MBE by measuring the oxidation of a non-fluorescent probe (2′,7′-dichlorodihydrofluorescein diacetate (DCFH-DA)) to its reduced fluorescent form (2′,7′-dichlorofluorescein (DCF)).

**Figure 5:**
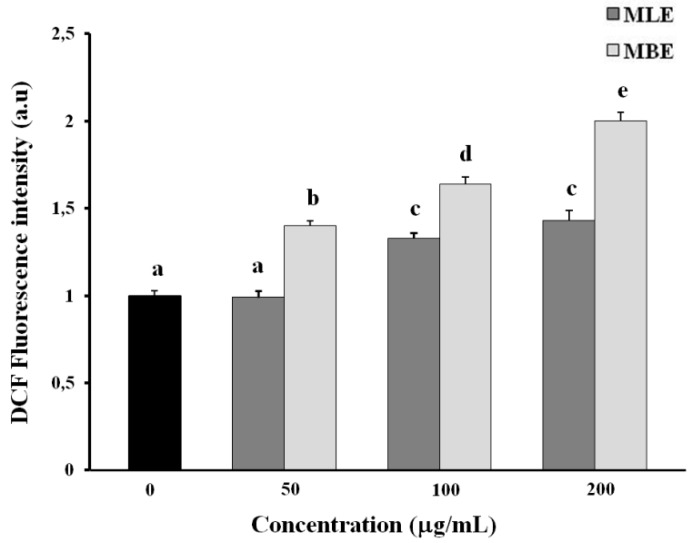
Quantitative evaluation of intracellular reactive oxygen species (ROS) production. HepG2 cells were incubated for 3 h with different concentrations (50–200 µM) of extracts and intracellular ROS was determined using the peroxide-sensitive fluorescent probe 2′,7′-dichlorodihydrofluorescein diacetate (DCFH-DA) as described in the Materials and Methods section. Values are the mean ± SD of three replicates from three independent experiments. Significant differences (*p* < 0.05) are highlighted with different letters.

Figure 5 shows higher ROS levels in MBE treated cells than MLE treated cells, according to the stronger apoptotic activity of MBE.

**Figure 6:**
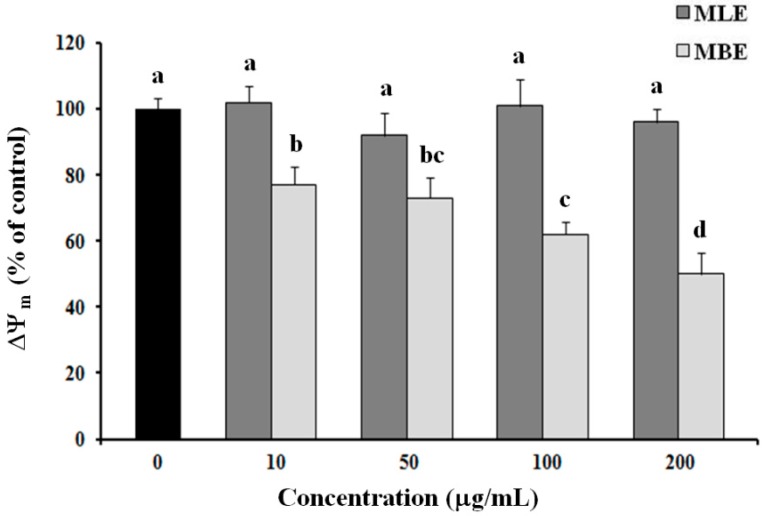
Measurement of mitochondrial membrane potential (ΔΨ_m_). The integrity of mitochondrial membranes in extract-treated cells was investigated after 3 h of treatment by measuring tetramethylrhodamine methyl ester (TMRM) fluorescence intensity. Change in ΔΨ_m_ was determined by flow cytometry. Values are the mean ± SD of three replicates from three independent experiments. Significant differences (*p* < 0.05) are highlighted with different letters.

Since excessive oxidative stress could lead to mitochondrial damage, causing a loss of mitochondrial membrane potential (ΔΨ_m_) and mitochondria-mediated apoptosis, we evaluated mitochondrial membrane polarization in HepG2 cells treated for 3 h with different concentrations of MLE and MBE; the cationic fluorescent probe tetramethylrhodamine methyl ester (TMRM), which is easily incorporated into mitochondria of viable cells, was used. As shown in Figure 6, MBE induced a remarkable decrease in mitochondrial membrane potential (ΔΨ_m_), leading to a decrease in TMRM fluorescence of about 50% at a concentration of 200 µg/mL, while MLE did not alter ΔΨ_m_ significantly.

Based on these results, MBE was chosen for further investigation in subsequent experiments.

### 2.7. Apoptosis Analysis by Western Blotting

**Figure 7:**
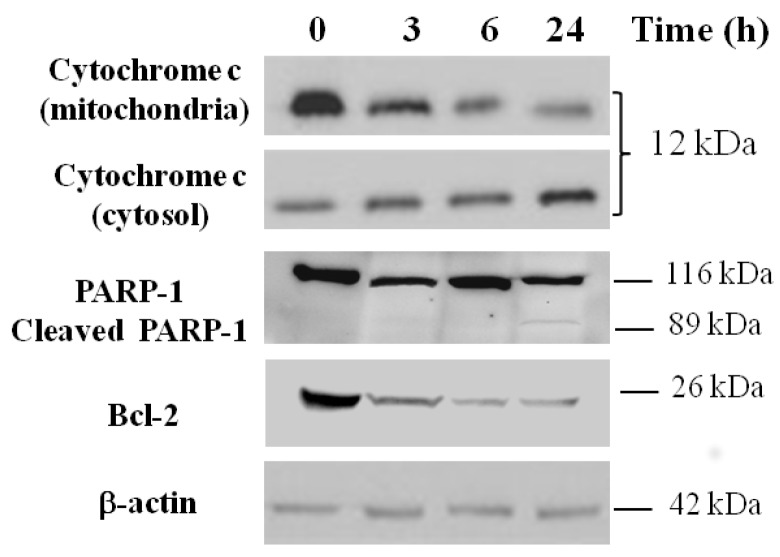
Western blot analysis of cytochrome c release, PARP-1 cleavage, and Bcl-2. HepG2 cells were incubated for 3, 6, and 24 h with 180 µg/mL (IC_50_) of methanol bark extract of *S. birrea.* For the detection of cytochrome c, cytosolic and mitochondrial fractions were prepared as previously described. Whole lysates were incubated with anti-PARP or anti-Bcl-2. β-actin was used as a loading control.

Mitochondria play a key role in apoptosis by releasing cytochrome c into the cytosol where it participates in activating caspase cascades. Therefore, we analyzed the intracellular distribution of this protein in HepG2 cells treated with 180 µg/mL of bark extract (IC_50_ value) for 3, 6, and 24 h. Immunoblot analysis highlights a progressive decrease in the mitochondria-enriched heavy membrane (HM) fractions and a consequent increase in the cytosolic fractions of cytochrome c in HepG2 treated cells vs. untreated control cells (Figure 7).

Moreover, Figure 7 also shows a significant time-dependent reduction of Bcl-2 (an anti-apoptotic protein localized on the outer mitochondrial membrane) levels in HepG2 cells treated with 180 µg/mL of bark extract vs. untreated control cells and the activation of caspase-3, detectable by the presence of the cleaved caspase substrate poly-ADP ribose polymerase (PARP-1) after 24 h of treatment.

## 3. Discussion

Nowadays, living organisms are repeatedly exposed to several oxidizing agents (some necessary for life) and it is well known, in fact, that an increased consumption of antioxidant-rich foods and food supplements are obtained often from natural sources. Antioxidants are molecules having several chemical structures that can act as inhibitors or quenchers of free radical reactions, delaying or inhibiting cellular damage. Plant species can be considered as a rich source of antioxidant molecules. *Sclerocarya birrea* is used by local populations to treat several ailments, as mentioned above, and scientific evidence supporting its common use is increasing. In this study, the in vitro antioxidant activity and the cytotoxic effects of methanol extracts from bark and leaves of *S. birrea* were investigated.

Phenolic compounds are the major constituents of *S. birrea* extracts [14,15,16] and methanol extracts of leaves and bark are reported to have the highest content of polyphenols compared to other extracts, as showed in Table 1; in particular, MLE was found to be rich in flavonoids, whereas MBE has the highest content of tannins. As natural antioxidants, flavonoids and tannins play an important role in scavenging free radicals and preventing degenerative diseases such as cardiovascular disease [1,2,3]; however, they are also involved in the antiproliferation of carcinogenic cells, in cell cycle regulation, in the induction of apoptosis, and in the inhibition of platelet aggregation, and also they have antibacterial, anti-inflammatory, and antiallergic properties [18,19,20,21].

In the present study, the radical scavenging activity was performed by in vitro BCB, ABTS^•+^, O_2_^−•^, and NO^•^ assays. Our results have shown that both of the investigated extracts (MLE and MBE) have a dose-dependent activity, similar to the reference standards (Figure 1). The comparison between the tested extracts showed that MBE has the highest radical scavenging activity than either the MLE or the standards (Trolox and ascorbic acid) (ABTS^•+^, O_2_^−•^, and NO^•^ assays). The lipid peroxidation of both extracts by the β-carotene bleaching assay (BCB) led to a lower value than the standard (BHT). Phenolic compounds including tannins and flavonoids are known as hydrophilic antioxidants and in aqueous systems they have the highest activity. However, BCB instead provides information about the level of lipophilic compounds [22].

The cytotoxic effect of methanol extracts from *S. birrea* leaves and bark were tested by the Calcein AM assay. HepG2 and normal fibroblast cells were treated with different concentrations of MLE and MBE for 24 h and the cells’ viability decreased in a dose-dependent manner. MBE had the highest cytotoxicity in HepG2 cells, whereas both extracts showed an IC_50_ value higher than 400 µg/mL in fibroblasts, demonstrating their potential anticancer effect. The higher cytotoxic activity of MBE was confirmed through more pronounced alterations in cellular morphology (Figure 3) such as vacuolization, cell detachment, and the presence round and translucent cells, which occurred at a lower concentration in bark extract than leaf extract treatment. Even in this case, the different compositions of the extracts can justify the different cellular responses to the treatments.

Cellular morphological changes can be considered as the key evidence of cytotoxicity to natural compounds or plant extracts, along with metabolic dysfunctions, alterations in proliferation rate, differentiation processes, and apoptosis. Apoptosis is a programmed cell death that plays a crucial role in embryonic development and maintenance of tissue homeostasis during adulthood by eliminating unnecessary or injured cells. Dysregulation of the apoptotic pathways results in a variety of diseases including the development and progression of some cancers [23,24]. Several studies have shown that a wide variety of plant extracts and natural substances can induce apoptosis in different tumor cells [25,26,27,28] and this occurrence can be exploited as a strategy against the progression of a tumor. The effects of MBE and MLE in inducing apoptotic processes were studied by flow cytometry and after 24 h of incubation, both extracts induced higher HepG2 cell death compared with untreated cells; however, bark extract treatment was more effective in inducing apoptosis in the cells, again suggesting a different effect of the various components of the extracts.

Accumulation of ROS and loss of mitochondrial membrane potential play an important role in apoptosis. ROS is physiologically generated in humans by aerobic respiration and too high a level of these species leads to oxidative stress in the cell, destroying membrane integrity and causing DNA damage. Incubation of HepG2 cells with *S. birrea* extracts for 3 h showed an increased level of ROS with both extracts, but ROS production was more pronounced when cells were treated with bark extract (Figure 5). MBE and MLE also decreased the mitochondrial membrane potential in a dose-dependent manner, particularly with MBE treatment. This loss of potential is probably due to the effect of the high levels of ROS.

Changes in mitochondrial membrane potential and mitochondrial permeability result in the release of cytochrome c from the mitochondria into the cytosol [29], a specific event in the early apoptosis mechanism. This process is blocked by Bcl-2, an anti-apoptotic protein localized on the outer mitochondrial membrane [30]. Finally, the cleaved form of the PARP-1, a physiological substrate of the activated protease caspase-3, is also considered a hallmark of apoptosis [31]. Caspase activation is a crucial process for apoptosis induction in cancer cells. Understanding the mechanism by which the anticancer drugs induce apoptosis may provide important information for proposing more effective anticancer treatments. Therefore, in order to clarify the cellular mechanisms by which bark extract of *S. birrea* induces cell death, mitochondrial apoptotic markers were analyzed by Western blot analysis. As shown in Figure 7, HepG2 cells incubated for 24 h with MBE showed a significant increase in cytochrome c release into the cytosol; the expression of the antiapoptotic protein Bcl-2 decreased over time, and PARP-1 cleavage was activated. Thus, these hallmarks suggest that MBE cellular treatment induces cell death through an intrinsic apoptotic pathway, probably triggered by ROS generation.

Overall, considering the obtained results, the effects of cellular exposure to MBE clearly demonstrates both a higher radical scavenging activity and a greater cytotoxicity of this extract compared to MLE. This difference could be explained by the different phytochemical contents. Indeed, previous studies have reported the chemical composition of bark and leaf extracts using HPLC-MS analysis [14,16]. Phenolic compounds such as flavonoid glycosides (myricetin 3-*O*-α-l-rhamnopyranoside, quercetin 3-*O*-β-d-glucopyranoside, quercetin 3-*O*-arabinoside, quercetin 3-*O*-α-l-rhamnopyranoside, kaempferol 3-*O*-α-l-rhamnopyranoside) and galloylated glycosides of quercetin and kaempferol were identified mainly in leaf extracts, whereas procyanidins (condensed tannins), including epicatechin 3-*O*-gallate, epigallocatechin3-*O*-gallate, and galloylepicatechin-epigallocatechin-3-*O*-gallate, were found in bark extracts of *S. birrea.* These results agree with spectrophotometric data reported in this work, where the flavonoid content (TFC) was dominant in leaves, whereas tannins (TTC) were abundant in bark.

The classes of flavonoids and procyanidins include a wide range of molecules with several chemical structures that affect their biological activity. As previously reported [32], the degree of polymerization of procyanidins enhances their effectiveness against radical species: indeed, dimers and trimers of procyanidins were found to be more active than monomeric flavonoids. Furthermore, the antioxidant properties such as the radical-scavenging activity of flavonoids decrease proportionally to an increase in the number of glycosidic moieties and also blockage of the C-3 hydroxyl group results in a total loss of antioxidant activity [33]. Not least, the glycosylation of some flavonoids seems to decrease their antiproliferative property [34]. It was also reported that cytotoxicity increases with the degree of polymerization and the percentage of galloylation of procyanidins and the gallate group seems to interfere with crucial cell functions; the galloylation appears to be a crucial structural feature defining the activity and toxicity of phenolic mixtures [35]. The cytotoxic activity of procyanidins in different types of cancer is, finally, well documented [36,37,38,39,40]. Therefore, these considerations agree with the results shown in this paper, justifying both the greater in vitro antioxidant activity and the most effective antiproliferative action seen for MBE. These data are in perfect agreement with those obtained in our previous work with the *S. birrea* root extract in which the content of procyanidins was very high [12]. Our findings allow us to suggest their constant use as a relatively new and promising strategy to prevent cancer.

Further studies will be directed to identifying the compounds responsible of the biological activities.

## 4. Materials and Methods

### 4.1. Chemicals

Folin–Ciocalteu reagent, sodium carbonate (Na_2_CO_3_), aluminum chloride (AlCl_3_), sodium nitrate (NaNO_3_), sodium hydroxide (NaOH), bovine serum albumin (BSA), sodium dodecyl sulfate (SDS), triethanolamine, iron(III) chloride (FeCl_3_), ABTS (2,2′-azino-bis(3-ethylbenzothiazoline-6-sulfonic acid)), potassium persulfate, β-carotene, linoleic acid, Tween 20, ascorbic acid, sodium nitroprusside (SNP), sulfanilamide, naphthylethylenediamine, nicotinamide adenine dinucleotide (NADH), phenazinemethosulfate (PMS), nitroblue tetrazolium (NBT), potassium phosphate monobasic (KH_2_PO_4_), gallic acid, quercetin, tannic acid, 6-hydroxy-2,5,7,8-tetramethylchroman-2-carboxylic acid (Trolox), butylated hydroxytoluene (BHT), Dulbecco’s Modified Eagle Medium (DMEM), dimethyl sulfoxide (DMSO), Calcein AM was purchased from Sigma Aldrich (Milan, Italy). All solvents were purchased from Carlo Erba Reagents (Milan, Italy). Trypsin-EDTA solution, FBS, glutamine, penicillin-streptomycin, and PBS were purchased from Euroclone (Milan, Italy). Tetramethylrhodamine methyl ester (TMRM, Life Technologies, Monza, Italy) was a kind gift from Massimo Lasorsa (IBBE, CNR, Bari, Italy).

### 4.2. Plant Extracts

Dried leaves and bark of *Sclerocarya birrea* were extracted as reported previously by Russo et al. (2013) [14] obtaining four extracts: *n*-hexane (HE), chloroform (CE), chloroform-methanol 9:1 (CME), and methanol (ME).

### 4.3. Total Content of Polyphenols, Flavonoids, and Tannins

The total content of polyphenols (TPC), flavonoids (TFC), and tannins (TTC) were evaluated using three different spectrophotometric assays, as reported by Armentano, et al. (2015) [12]. TPC was expressed as mg gallic acid equivalent (GAE)/g of dried extract, TFC was expressed as mg of quercetin equivalent (QE)/g of dried extract, and TTC as mg of tannic acid equivalent (TAE)/g of dried extract.

### 4.4. In Vitro Antioxidant Activity

#### 4.4.1. ABTS Test

The free radical scavenging capacity of each plant extract was studied using the 2,2′-azino-bis(3-ethylbenzothiazoline-6-sulfonic acid) diammonium salt (ABTS) radical assay. Experiments were performed according to the previous report [12] with slight modifications. ABTS (7 mM) and potassium persulfate (2.45 mM) solutions were mixed and then the mixture was allowed to stand in the dark at room temperature for 16 h before use in order to produce the ABTS radical (ABTS^•+^). For the analysis of the extracts, the ABTS radical solution was diluted with distilled water to an absorbance of 1.00 at 734 nm. Extracts were tested at different concentrations (0–200 µg/mL) and the results were expressed as the percentage of radical inhibition and then converted to IC_50_, which is the concentration of sample required to inhibit 50% of the ABTS radicals. Trolox was used as the reference standard.

#### 4.4.2. BCB Assay

The inhibition of lipid peroxidation was monitored by the β-carotene/linoleic acid system. The assay is based on the discoloration of β-carotene, which is caused by an attack of the lipid peroxide radicals (LOO^•^) that is generated by the oxidation of linoleic acid; the addition of an effective antioxidant reduces the discoloration, which can be quantified spectrophotometrically at 470 nm. BHT was used as a positive control and the results were expressed as the percentage of antioxidant activity (% AA) [41].

### 4.5. Nitric Oxide (NO) Scavenging Activity

Nitric oxide scavenging activity can be estimated by using the Griess reaction. The compound sodium nitroprusside is known to decompose in aqueous solution at physiological pH 7.2, producing NO, which is able to react (under aerobic conditions) with oxygen to produce stable products (nitrate and nitrite) that can be determined using the Griess reagent. Scavengers of NO compete with oxygen and leads to reduced production of nitrite ions as reported by a previous study [18]. Results were expressed as % of inhibition.

### 4.6. Superoxide Anion (O_2_^−•^) Scavenging Activity

Measurement of superoxide anion scavenging activity of *S. birrea* extracts was determined as previously reported [18]. Superoxide radicals are generated in PMS-NADH systems by oxidation of NADH and assayed by the reduction of nitroblue tetrazolium (NBT). l-Ascorbic acid was used as a control. The decreased absorbance of the reaction mixture indicates increased superoxide anion scavenging activity. Results were expressed as the percentage inhibition of superoxide anion generation and it was calculated using the following formula:

% Inhibition = [(A_0_ − A_1_)/A_0_] × 100 (1)

where A_0_ is the absorbance of the control and A_1_ is the absorbance of the extract and standards.

### 4.7. Cell Culture and Treatment with Extracts

Human hepatoma (HepG2) and normal human dermal fibroblast (adult, HDFa, Life Technologies) cell lines were cultured and maintained under the same conditions as previously reported [12]. Bark and leaf methanol extracts (MBE and MLE, respectively) were dissolved in DMSO at the stock solution of 50 mg/mL and then diluted to the required concentrations. The final DMSO concentration in the cultures was no greater than 0.8%: this concentration had no effect on cell viability. DMSO-treated cells were used as controls in all the experiments. Both cell lines were treated when they were 60–70% confluent, at passages 4 to 10 (HepG2) and 4 to 8 (HDF).

### 4.8. Cytotoxicity Analysis

Leaf and bark methanol extracts of *S. birrea* were tested against HepG2 and normal human dermal fibroblast cell lines using the Calcein AM viability assay. Cells were seeded in 96-well black-walled plates (1 × 10^4^ cells/ well) and after 24 h were incubated with various concentrations of MBE or MLE (10, 50, 100, 200, and 300 µg/mL) at 37 °C for 24 h. Human fibroblasts were incubated with up to 400 µg/mL of MBE/MLE. Thereafter, the media were discarded from the wells and the cells were incubated with 100 µL of 1 µM Calcein AM in PBS for 30 min at 37 °C. Cellular Calcein fluorescence was detected using a plate reader (GLOMAX Multidetection System, Promega, Madison, WI, USA) at excitation and emission wavelengths of 490 and 510–570 nm, respectively.

### 4.9. Cell Imaging

HepG2 cells were cultured in standard conditions in 24-well tissue culture plates at a seeding density of 2 × 10^5^ cells/well and treated with increasing concentrations (10, 50, 100, and 200 µg/mL) of MBE and MLE for 24 h. The untreated cells served as a control. Changes in cell morphology were imaged using inverted phase contrast microscopy at 40× magnification (Nikon Eclipse TS100, Nikon, Tokyo, Japan).

### 4.10. Apoptosis Assay

The percentage of apoptotic cells was determined using the Annexin V/7-AAD double staining assay, according to the manufacturer’s protocol (BD Pharmingen, San Jose, CA, USA). In brief, HepG2 cells were plated in a 12-well culture plate at a density of 2 × 10^5^ cells/well and treated with MBE and MLE (50, 100, and 200 µg/mL) for 24 h. At the end of the treatment, cells were harvested and suspended in 500 µL of binding buffer containing 5 μL Annexin V and 5 μL 7-AAD and then incubated in the dark for 15 min. Finally, at least 1 × 10^4^ stained cells per sample were analyzed in a BD FACSCanto II (BD Pharmingen, San Jose, CA, USA) flow cytometry analyzer.

### 4.11. Reactive Oxygen Species (ROS) Measurements

The intracellular ROS level was measured using a fluorescent probe, 2′,7′-dichlorodihydrofluorescein diacetate (DCFH-DA), as previously reported [12]. Briefly, HepG2 cells were seeded into dark 96-well tissue culture plates at a density of 5 × 10^4^ cells/well and incubated for 24 h at 37 °C. Following treatment with MLE or MBE (50, 100, and 200 µg/mL) for 3 h, cells were stained with 10 µM DCFH-DA for 30 min at 37 °C in the dark. The fluorescence was measured by the GLOMAX Multidetection System (Promega, Madison, WI, USA) using a blue filter (Ex 490 nm, Em 510–570 nm).

### 4.12. Mitochondrial Membrane Potential (MMP) Evaluation

Changes in mitochondrial membrane potential (MMP) were monitored by flow cytometry (FACSCanto II) with the mitochondrial tracking fluorescent dye TMRM. In brief, 2 × 10^5^ cells were cultured in 12-well plates and treated with MLE and MBE at different concentrations (10, 50, 100, and 200 µg/mL) for 3 h. The cells were then detached, washed in ice-cold PBS, and incubated for 20 min at 37 °C in darkness with 150 nM TMRM in PBS. After dilution with PBS, cells were analyzed by FACS with an excitation wavelength of 488 nm and an emission wavelength of 575 nm.

### 4.13. Western Blot Analysis

HepG2 cells were exposed to an IC_50_ concentration of MBE (180 µg/mL) for 3, 6, and 24 h and then harvested and washed twice with ice-cold PBS. Mitochondria enrichment was obtained as previously described [12]. The membranes were incubated overnight at 4 °C with the specific primary antibody (anti-cytochrome c, 1:2000 Abcam). Moreover, whole lysate was blotted onto a nitrocellulose membrane and the nonspecific binding sites were blocked with TBST buffer containing 5% nonfat dry milk. The membranes were incubated with anti-PARP-1 or anti-Bcl-2, both diluted 1:200 (Santa Cruz Biotech, Dallas, TX, USA). After incubation with appropriate secondary antibodies, detection was performed using the enhanced chemiluminescence (ECL) kit (GE).

### 4.14. Statistical Analysis

All the results are presented as mean ± SD of three independent experiments performed in triplicate. In the viability assays, the percentage survival values were normalized by an arcsine square root transformation and then compared using analysis of variance (ANOVA) and Tukey’s HSD test. In the measurement of reactive oxygen species and mitochondrial membrane potential, statistical significances were analyzed by one-way analysis of variance (ANOVA) and Tukey’s HSD test. Both analyses were performed using software R version 2.8.1 (R Development Core Team, Vienna, Austria, 2008). Different letters denote significant differences (*p* < 0.05).

## Figures and Tables

**Table 1:** Total polyphenol content (TPC), total tannin content (TTC), and total flavonoid content (TFC) in leaf and bark extracts of *S. birrea.*

	TPC (mg GAE/g)	TTC (mg TAE/g)	TFC (mg QE/g)
HLE	30.2 ± 1.3	102.4 ± 3.1	8.3 ± 1.2
CLE	41.7 ± 0.6	175.8 ± 5.5	17.7 ± 2.0
CMLE	49.9 ± 3.7	74.2 ± 5.5	31.1 ± 4.1
MLE	62.6 ± 0.8	90.2 ± 4.7	132.7 ± 10.4
HBE	79.9 ± 0.7	196.1 ± 12.5	4.7 ± 0.5
CBE	31.9 ± 1.6	211.2 ± 14.8	9.5 ± 1.2
CMBE	31.3 ± 0.2	158.7 ± 1.6	15.6 ± 2.1
MBE	241.3 ± 8.5	949.5 ± 29.7	57.7 ± 3.5

(GAE)/g = milligram gallic acid equivalent per gram of dried extract; (QE)/g = milligram of quercetin equivalent per gram of dried extract; (TAE)/g = milligram of tannic acid equivalent per gram of dried extract; HLE = *n*-hexane extract of leaves; CLE = chloroform extract of leaves; CMLE = chloroform/methanol extract of leaves; MLE = methanol extract of leaves; HBE = *n*-hexane extract of bark; CBE = chloroform extract of bark; CMBE = chloroform/methanol extract of bark; MBE = methanol extract of bark.
